# Development of a Novel, Single-Cycle Replicable Rift Valley Fever Vaccine

**DOI:** 10.1371/journal.pntd.0002746

**Published:** 2014-03-20

**Authors:** Shin Murakami, Kaori Terasaki, Sydney I. Ramirez, John C. Morrill, Shinji Makino

**Affiliations:** 1 Department of Microbiology and Immunology, the University of Texas Medical Branch, Galveston, Texas, United States of America; 2 Department of Pathology, the University of Texas Medical Branch, Galveston, Texas, United States of America; 3 Center for Biodefense and Emerging Infectious Diseases, the University of Texas Medical Branch, Galveston, Texas, United States of America; 4 UTMB Center for Tropical Diseases, the University of Texas Medical Branch, Galveston, Texas, United States of America; 5 Sealy Center for Vaccine Development, the University of Texas Medical Branch, Galveston, Texas, United States of America; Centers for Disease Control and Prevention, United States of America

## Abstract

Rift Valley fever virus (RVFV) (genus *Phlebovirus*, family *Bunyaviridae*) is an arbovirus that causes severe disease in humans and livestock in sub-Saharan African countries. Although the MP-12 strain of RVFV is a live attenuated vaccine candidate, neuroinvasiveness and neurovirulence of MP-12 in mice may be a concern when vaccinating certain individuals, especially those that are immunocompromised. We have developed a novel, single-cycle replicable MP-12 (scMP-12), which carries an L RNA, M RNA mutant encoding a mutant envelope protein lacking an endoplasmic reticulum retrieval signal and defective for membrane fusion function, and S RNA encoding N protein and green fluorescent protein. The scMP-12 underwent efficient amplification, then formed plaques and retained the introduced mutation after serial passages in a cell line stably expressing viral envelope proteins. However, inoculation of the scMP-12 into naïve cells resulted in a single round of viral replication, and production of low levels of noninfectious virus-like particles. Intracranial inoculation of scMP-12 into suckling mice did not cause clinical signs or death, a finding which demonstrated that the scMP-12 lacked neurovirulence. Mice immunized with a single dose of scMP-12 produced neutralizing antibodies, whose titers were higher than in mice immunized with replicon particles carrying L RNA and S RNA encoding N protein and green fluorescent protein. Moreover, 90% of the scMP-12-immunized mice were protected from wild-type RVFV challenge by efficiently suppressing viremia and replication of the challenge virus in the liver and the spleen. These data demonstrated that scMP-12 is a safe and immunogenic RVFV vaccine candidate.

## Author Summary

Rift Valley fever virus (RVFV) is a mosquito-borne zoonotic pathogen, which causes febrile illness, encephalitis and fatal hemorrhagic fever in humans and severe hepatic disease with high mortality and spontaneous abortion rates in ruminants. RVFV is endemic to the African continent. Because many different mosquito species support RVFV replication, the virus has the potential to spread to other areas of the world, such as North and South America, Asia, and Europe and could cause serious public health problems and economic losses. Consistent with this concern, RVFV has caused epidemic disease in the Arabian Peninsula. Currently, there is no approved vaccine suitable for mass vaccination programs of humans. Although the MP-12 strain of RVFV is a live attenuated vaccine candidate, its neuroinvasiveness and neurovirulence in mice are areas of concern, especially when considering the immunization of children and the immunocompromised. In this study, we present a novel MP-12-based, single-cycle replicable RVF vaccine candidate. This vaccine candidate was not neurovirulent in mice and was effective in protecting immunized mice from wild-type RVFV challenge, indicating its potential to be developed as a safe vaccine for use in both humans and livestock.

## Introduction

Rift Valley fever virus (RVFV), a member of the genus *Phlebovirus* within the family *Bunyaviridae*, carries a tripartite, single-stranded and negative–sense RNA genome [1]–[3]. The L RNA encodes the L protein, a viral RNA-dependent RNA polymerase; the M RNA encodes four proteins, including two accessory proteins, the NSm and 78-kDa proteins, and two major viral envelope proteins, Gn and Gc (Gn/Gc); and the S RNA uses an ambisense strategy to express the N protein and an accessory protein, NSs. In infected cells viral RNA synthesis occurs in the cytoplasm, while viral assembly and budding take place at the Golgi apparatus, where Gn/Gc accumulates.

The virus is transmitted by mosquitoes and is maintained in nature, in sub-Saharan Africa, at least in part, by transovarial transmission. RVFV is able to infect various species of mosquitoes [4] and has the potential to spread to other areas of the world. Indeed, RVFV has already spread outside of the African continent to the Arabian Peninsula. The intentional spread of RVFV is also a serious national biosecurity concern. Human infection usually results in febrile illness, but may also cause viral hemorrhagic syndrome, encephalitis, and ocular disease [5]–[7]. RVFV also infects domestic ruminants and causes high mortality and spontaneous abortion rates with severe hepatic disease [8]. Introduction of RVFV to other areas of the world, including North and South America, Asia, and Europe, could cause serious public health problems and economic losses.

RVFV spread can be prevented by the effective vaccination of animals and humans [1]. RVFV is considered to be serologically monotypic [9]–[11], and humoral immunity, particularly neutralizing antibodies that recognize Gn/Gc, is important for protection [12]–[20]. Although a good human RVFV vaccine is urgently needed, there is no approved vaccine that can be adapted to massive vaccination programs. The MP-12 strain of RVFV [21], which was developed by the serial passage of wild-type (wt) RVFV strain ZH548 in the presence of the mutagen 5-fluorouracil, is markedly attenuated and yet retains its immunogenicity [22]–[28]; hence, MP-12 is a promising live vaccine candidate for both human and veterinary use. However, intraperitoneal (i.p.) inoculation of young mice with MP-12 can result in efficient virus replication in the central nervous system (CNS) (J. Morrill et al, unpublished data). Furthermore, i.p. inoculation of SCID mice with MP-12 results in the development of neurological signs and death of all mice [29]. These data suggest that MP-12 can invade the CNS and undergo efficient replication in immunocompromised animals, and may potentially do so in immunocompromised humans as well. However, neurovirulence tests in rhesus macaques show MP-12 to be less neuroinvasive and neurovirulent than acceptable lots of yellow fever or measles vaccine (28). Even so, neuroinvasiveness and neurovirulence is of concern when considering RVFV immunization of the general public, given the diversity of ages, health statuses and genetic backgrounds. Thus, it is important to develop highly immunogenic RVFV vaccines with reduced or no neurovirulence.

To develop a safe and immunogenic RVF vaccine, we have generated a novel, single-cycle replicable MP-12 (scMP-12), which does not cause systemic infection in immunized hosts, while resulting in expression of all viral structural proteins and production of noninfectious, virus-like particles (VLPs) in naïve cells infected with scMP-12. The scMP-12 did not show any sign of neurovirulence after intracranial inoculation into suckling mice, demonstrating its safety. scMP-12-immunized mice elicited neutralizing antibodies and were efficiently protected from wt RVFV challenge by inhibiting wt RVFV replication in various organs and viremia. Our data suggest that scMP-12 has excellent potential to be developed as a safe RVF vaccine.

## Materials and Methods

### Ethics statement

All mouse studies were performed in facilities accredited by the Association for Assessment and Accreditation of Laboratory Animal Care in accordance with the Animal Welfare Act, NIH guidelines and U.S. federal law. The animal protocol was approved by the UTMB Institutional Animal Care and Use Committee. The wt RVFV ZH501 strain was used in an enhanced ABSL-3 laboratory within the Galveston National Laboratory at UTMB in accordance with NIH guidelines and U.S. federal law.

### Cells and viruses

Vero E6 cells and BSR-T7/5 cells [30], the latter of which stably express T7 RNA polymerase, were maintained as described previously [31], [32]. BHK-21 cells were maintained in minimal essential medium (MEM) α medium (Gibco) supplemented with 5% fetal bovine serum (FBS). The MP-12 strain of RVFV was generated by reverse genetics [31].

### Plasmid constructions and scMP-12 generation

A standard PCR-based method, in which pProT7-M encoding antiviral-sense M RNA [31] served as a template, was used to generate pProT7-M-Gn/GcΔ5, which expresses M-Gn/GcΔ5 RNA carrying a deletion between nucleotide positions 3597 and 3611 in the M segment. A Quickchange II site-directed mutagenesis kit (Agilent Technologies) was used to obtain pProT7-M-Gn/GcΔ5-derived mutants, each of which carried an amino acid substitution(s) within a putative fusion peptide. Plasmid pCAGGS-bla-G was constructed by inserting the Not I-EcoR V fragment of pCX4-bsr [33], which contains the encephalomyocarditis virus internal ribosomal entry site and blasticidin-resistant gene, into the Not I and Stu I sites of pCAGGS-G, which carries the entire open reading frame (ORF) of MP-12 M RNA encoding 78-KDa, NSm, Gn and Gc proteins. The sequences of all of the constructs were confirmed not to contain unwanted mutations. MP-12, scMP-12, and MP-12-based, 2-segmented virus replicon particles (VRP) were generated by using a reverse genetics system [31]. Briefly, BSR-T7/5 cells were co-transfected with plasmids encoding the L, N, and Gn/Gc proteins, and anti-viral sense L, M, and S RNAs for MP-12 recovery. scMP-12 recovery was performed by using a similar method with the following modifications: a plasmid expressing the S RNA carrying an N gene and green fluorescent protein (GFP) (S-GFP RNA) was used in place of that expressing the S RNA; a plasmid encoding M-Gn/GcΔ5 RNA with two amino-acid substitutions, F826N and N827A, was used in place of that expressing the M RNA; and a plasmid expressing the MP-12 Gn/Gc optimized for bovine codon usage was used in place of that expressing the MP-12 Gn/Gc to prevent or minimize homologous RNA recombination events between expressed mRNA encoding Gn/Gc and the replicating M RNA mutant. For VRP recovery, a plasmid expressing S-GFP RNA was used in place of the plasmid encoding the S RNA and the plasmid encoding the M RNA was eliminated. Culture fluid was collected at 5, 10 and 10 days post transfection for MP-12, scMP-12, and VRP, respectively.

### Generation of Vero-G cells

Vero E6 cells were transfected with pCAGGS-bla-G, and incubated in the presence of 20 µg/ml of blasticidin from 1 day post-transfection. After obtaining blasticidin-resistant cell clones by limiting dilution, each cell clone was tested for Gn protein expression by indirect immunofluorescence with an anti-Gn monoclonal antibody (R1-4D4) [34], and a cell clone expressing highest levels of Gn was selected and designated as Vero-G cells.

### Plaque assay

A standard plaque assay was used to determine the infectivity of MP-12 [31]. For determining the infectivity of scMP-12 and VRP, Vero-G cells in 6-well plates were inoculated with 400 µl of serially diluted samples and incubated for 1 h at 37°C. After removal of the inocula, cells were incubated with MEM containing 0.6% Tragacanth gum (MP Biomedicals), 5% FBS, and 5% tryptose phosphate broth at 37°C. After 3 days incubation, cells were washed with phosphate-buffered saline (PBS) and fixed with PBS containing 4% paraformaldehyde for 20 min at room temperature. After removing paraformaldehyde and overlays, the cells were permeabilized with 0.1% Triton-X100 and incubated with anti-N rabbit polyclonal antibody, which was generated by injecting a purified, bacterially-expressed fusion protein consisting of glutathione-S-transferase and full-length MP-12 N protein into rabbits, followed by incubation with horseradish peroxidase-conjugated, anti-rabbit IgG antibody. The plaques were visualized with Nova RED peroxidase substrate (Vector Laboratories, Burlingame, CA). This modified plaque assay was also used for observing plaque morphologies of MP-12 in Vero-G cells.

### Cell fusion assay

The cell fusion assay was performed as previously described [35], [36] with some modifications. Briefly, BSR-T7/5 cells were co-transfected with plasmids encoding the Venus, N, and L proteins, and M-Gn/GcΔ5 RNA or M-Gn/GcΔ5 RNA with single amino acid substitutions, and incubated at 37°C for 24 h. To initiate cell fusion, the cells were washed with Mg^2+^- and Ca^2+^-containing acidic PBS (pH adjusted to 5.2 with citric acid) and treated with the acidic PBS for 5 min., and then incubated in complete medium at 37°C for 60 min. GFP signals in the cells were observed under a fluorescence microscope (Zeiss).

### Indirect immunofluorescence assay

BSR-T7/5 cells were co-transfected with plasmids encoding the N and L proteins, and M-Gn/GcΔ5 RNA or its mutant. Twenty-four hours after transfection, cells were fixed with 4% paraformaldehyde and permeabilized with 0.1% Triton-X100, or not permeabilized. Cells were incubated with the primary monoclonal antibody that recognizes Gn (R1-4D4) or Gc (R1-5G2) [37] for 1 h at room temperature and with the Alexa-594-conjugated secondary antibody for 1 h at room temperature, and observed under a fluorescence microscope.

### Western blot analysis

Cells were harvested by using a cell scraper and washed with PBS. After incubation of the harvested cells on ice for 20 min in cell lysis buffer (20 mM Tris-HCl, 150 mM NaCl, 1% Triton X-100), the cell lysate was centrifuged at 2,000 rpm for 3 min by using a microcentrifuge. The resultant supernatant was mixed with the same amount of 2× sample buffer and boiled for 5 min. Equal amounts of samples were subjected to SDS-polyacrylamide gel electrophoresis. Proteins were electroblotted onto polyvinylidene difluoride membranes (Millipore). After blocking the membrane with 1% bovine serum albumin for 1 h, the membranes were incubated with the primary antibody for 1 h at room temperature. After incubation with the secondary antibody for 1 h at room temperature, the blots were developed by using an ECL kit (GE Healthcare). Anti-MP-12 mouse polyclonal antibody [31] was used to detect the virus-specific proteins.

### Northern blot analysis

Total RNAs were extracted by using TRIzol reagent (Invitrogen) and subjected to Northern blot analysis as described previously [38]. Viral-sense-specific, digoxigenin-labeled RNA probes [31] and a digoxigenin system (Roche) were used for the detection of viral RNAs. The L RNA probe hybridizes with viral-sense L RNA at nucleotide positions 19–756, the M RNA probe at nucleotide positions 1297–2102, and the S RNA probe at nucleotide positions 39–776 from the 3′ ends of the viral-sense RNA segments. The probe that hybridizes with anti-viral sense S RNA binds at nucleotide positions 39–776 from the 5′ end of the anti-viral-sense S RNA segment.

### Virus purification

Culture medium harvested from plasmid-transfected cells or scMP-12-infected cells was clarified by centrifugation at 3,000 rpm for 15 min by using a tabletop centrifuge. The clarified supernatant was layered on top of a step sucrose gradient consisting of 20, 30, 50, and 60% sucrose (wt/vol) and centrifuged for 3 h at 26,000 rpm at 4°C using a Beckman SW28 rotor [32]. The particles at the interface of 30 and 50% sucrose were collected, diluted and subjected to a second sucrose gradient centrifugation consisting of 20, 30, 50, and 60% sucrose for 18 h at 4°C. The particles at the interface of 30 and 50% sucrose were collected and pelleted down through a 20% sucrose cushion at 38,000 rpm for 2 h at 4°C using a Beckman SW41 rotor.

### Serial passage of the scMP-12 in Vero-G cells

scMP-12 was serially passaged 10 times in Vero-G cells under the following three conditions for each passage: inoculation without sample dilution and harvest at 4 days p.i., inoculation after 10 times sample dilution and harvest at 5–6 days p.i., and inoculation after 100 times sample dilution and harvest at 7 days p.i. We visually inspected for an increase in the number of GFP-positive cells every day. Each of the culture fluids collected was also inoculated into Vero E6 cells, and the GFP signal was examined daily up to 5 days p.i.

### Experimental infection for scMP-12 safety test

Two-day-old CD1 mice were intracranially inoculated with 10^4^ PFU of MP-12, scMP-12, or the same volume of Hank's balanced salt solution (HBSS). We monitored the mice for survival for 21 days.

### Immunization and protection

CD1 mice (5-week-old females) were intramuscularly immunized with 10^4^ PFU of MP-12, 10^5^ PFU of MP-12, 10^5^ PFU of scMP-12, or 10^5^ PFU of VRP. Thirty-six days later, blood was collected from the retro-orbital venous plexus of the mice. Forty days after vaccination, the immunized mice were challenged subcutaneously with 10^3^ PFU of the virulent RVFV strain ZH501, which was equivalent to approximately 1,000 times the 50% minimal lethal dose (LD_50_). The animals were observed for survival and clinical signs of disease for 21 days post-challenge. To determine the effect of immunization on virus replication, sera and specimens of liver, spleen and brain were harvested from randomly selected animals at 3, 6, 9 and 11 days post-challenge. Sera and 10% tissue homogenates were tested for virus presence and titer in Vero E6 cells, as previously described (39).

### Virus neutralization assay

Serum neutralizing antibody titers were determined by using an 80% plaque-reduction neutralization test (PRNT80), as previously described [39].

## Results

### An outline of the scMP-12 system

**Figure 1:**
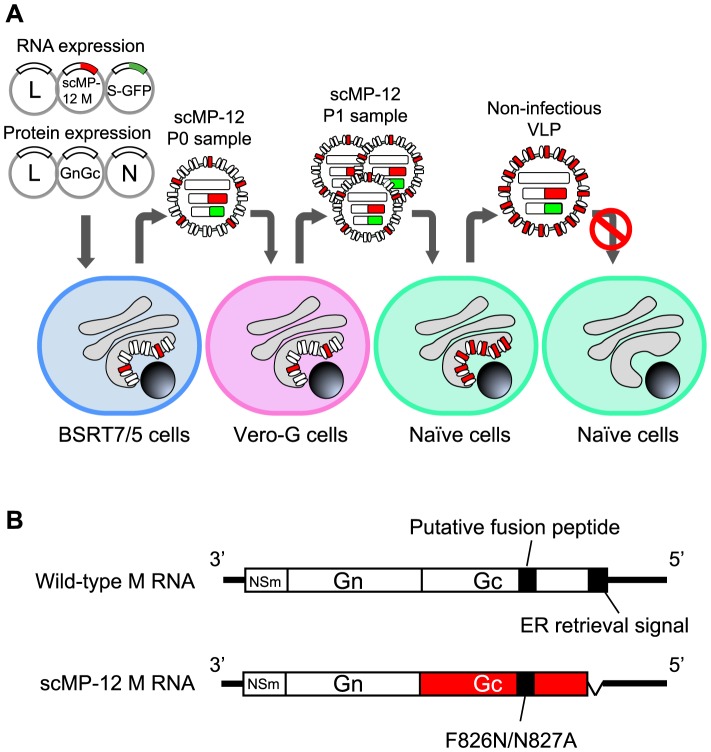
Schematic diagram of the MP-12-based scMP-12 system. (A) scMP-12 was generated in BSR-T7/5 cells stably expressing T7 polymerase by co-transfection of plasmids, which expressed the L, N, and Gn/Gc proteins, as well as the L RNA, S-GFP RNA and scMP-12 M RNA encoding Gc mutant. scMP-12 contains viral RNAs and is competent for initiating infection, as it carries wt Gn/Gc derived from the protein expression plasmid. scMP-12 is further propagated in Vero-G cells stably expressing wt Gn/Gc. Inoculation of scMP-12 into naïve cells results in viral RNA synthesis, expression of viral proteins, and production of non-infectious VLPs. (B) Schematic diagram of antiviral-sense M RNA and scMP-12 M RNA. The ORFs of NSm and Gn genes are shown in white boxes, while the Gc gene ORF appears in the red box. The black bars represent both the putative fusion peptide and the ER retrieval signal. In scMP-12 M RNA, mutations within the putative fusion peptide and deletion of the ER retrieval signal are shown.

We designed the scMP-12 system as shown in Fig. 1A. scMP-12 carries a membrane-fusion defective mutant of Gn/Gc and is rescued by using a modified MP-12 reverse genetics system [31], in which BSR-T7/5 cells stably expressing T7 polymerase [30] are co-transfected with three RNA-expression plasmids expressing the L RNA, a mutant M RNA encoding a membrane-fusion defective mutant of Gn/Gc, and a S-GFP RNA encoding the N and GFP proteins, as well as three protein expression plasmids encoding the L, N, and Gn/Gc proteins. The scMP-12 that is produced is infectious due to the presence of Gn/Gc and undergoes amplification in Vero-G cells stably expressing Gn/Gc. Inoculation of the amplified scMP-12 into naïve cells results in viral RNA synthesis, expression of viral proteins, including L, N and the fusion-defective Gn/Gc, and production of noninfectious VLPs containing the fusion-defective Gn/Gc. In immunized hosts, scMP-12 undergoes single cycle replication in infected cells, resulting in the intracellular accumulation of all of the viral structural proteins and the production of noninfectious VLPs; scMP-12 particles in the inoculum, viral proteins accumulated in scMP-12-infected cells and released noninfectious VLPs all serve as immunogens to elicit immune responses to RVFV proteins. Due to its characteristic single-cycle replication, it is highly unlikely that the scMP-12 can cause systemic infection or invade the CNS of immunized animals or humans.

### Establishment of a fusion assay

**Figure 2:**
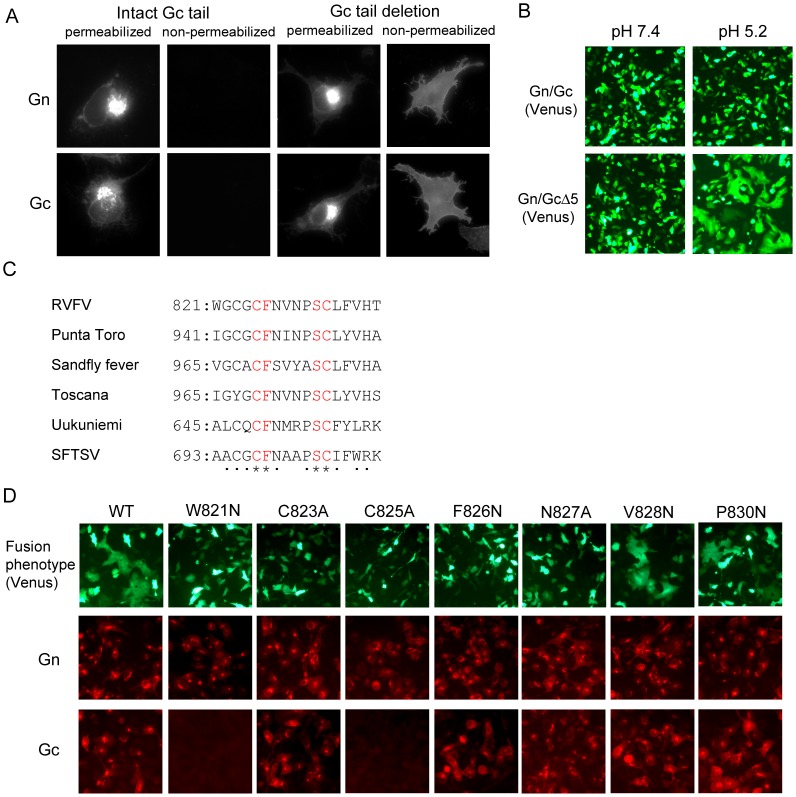
Development of a fusion assay and identification of amino residues within the putative fusion peptide whose mutation abolishes membrane fusion. (A) Effect of Gc cytoplasmic tail deletion on subcellular localization of Gn/Gc. BSR-T7/5 cells were transfected with plasmid encoding wt Gn/Gc or that encoding Gn/GcΔ5. At 24 h post-transfection, the cells were fixed, treated with Triton X-100 (permeabilized) or left without treatment (non-permeabilized) and stained with anti-Gn (Gn) or anti-Gc (Gc) monoclonal antibodies. (B) Syncytium formation by expressed Gn/GcΔ5. BSR-T7/5 cells were co-transfected with either plasmid encoding Gn/Gc or Gn/GcΔ5 in addition to that encoding the Venus protein. At 24 h post-transfection, cells were treated with either low-pH buffer (pH 5.2) or neutral buffer (pH 7.4) for 1 min, further incubated for 1 h, and observed under a fluorescence microscope. (C) Alignment of the putative fusion peptide of Phleboviruses. Asterisks and dots represent the conserved residues and relatively conserved residues, respectively. The numbers represent the location of the putative fusion peptide in the ORF encoded in the M RNA. SFTSV: severe fever with thrombocytopenia syndrome virus (D) Fusion activity of Gn/GcΔ5-based mutants, each carrying a single amino acid mutation within the putative fusion peptide, and their reactivity to Gn- or Gc-specific monoclonal antibodies. BSR-T7/5 cells were co-transfected with plasmids encoding the L, N, and Venus proteins and M RNA encoding Gn/GcΔ5 (WT) or a M RNA mutant encoding Gn/GcΔ5 and a single amino-acid substitution within the putative fusion peptide. Cells were treated with a low pH buffer and observed under a fluorescence microscope for the fusion assay (top row). The cells were also fixed and stained with anti-Gn monoclonal antibody (middle row) or anti-Gc monoclonal antibody (bottom row).

To isolate fusion-defective Gn/Gc mutants suitable for scMP-12, we first developed a cell-to-cell membrane fusion assay. Phlebovirus glycoprotein-induced, virus-cell membrane fusion requires a low pH (∼pH 5.4) environment [35]. Exposure of cells expressing RVFV Gn/Gc to low pH conditions does not induce cell-to-cell fusion due to the absence of Gn/Gc at the plasma membrane; RVFV Gn/Gc accumulates at the Golgi apparatus and the endoplasmic reticulum (ER) in infected cells and in expressed cells. Phleboviruses have an ER retrieval signal of ∼5 amino acids in the cytoplasmic tail of Gc [40], and removal of this signal in the Gc of Uukuniemi virus (a Phlebovirus) results in an accumulation of expressed Gn/Gc at the Golgi apparatus and plasma membrane [40]. Likewise, mutant MP-12 Gn/Gc lacking the terminal C-terminal 5-amino-acid residues of the Gc (Gn/GcΔ5) primarily accumulated at the Golgi apparatus when expressed, and some mutant glycoprotein was translocated to the plasma membrane (Fig. 2A). Exposure of the cells expressing Gn/GcΔ5, but not those expressing wt Gn/Gc, to low pH conditions induced cell-to-cell membrane fusion; fusion was not observed at neutral pH conditions for cells expressing Gn/GcΔ5 (Fig. 2B). These data suggest that Gn/GcΔ5 protein that localized to the plasma membrane was fusion-competent only under low pH conditions.

### Identification of key residue(s) for virus membrane fusion in Gc

We sought to generate fusion-defective Gn/Gc mutants by altering amino acids in the putative fusion peptide, which was previously predicted by computational studies and structural analysis [41], [42]. Alignment of the predicted fusion peptide sequences of several Phleboviruses revealed the presence of a highly conserved cysteine residue at position 825 (C825), which is involved in a disulfide bond in the Gc [41], and a phenylalanine residue at position 826 (F826) (Fig. 2C). Because hydrophobic residues are important for the insertion of fusion peptides into the cell plasma membrane [43], we tested the fusion competence of a series of Gn/GcΔ5-derived mutants, in which the F826 was changed to a hydrophilic residue, or its surrounding hydrophobic residues and hydrophilic residues were changed to hydrophilic residues or hydrophobic residues, respectively (Fig. 2D). While the V828N and P830N mutants retained fusion activity, the other mutants lost such activity (Fig. 2D). Anti-Gn monoclonal antibody recognized all of the Gn/Gc mutants, while the anti-Gc monoclonal antibody R1-5G2 failed to detect the W821N and C825A mutants, implying an alteration of the Gc conformation occurred from these mutations. From the C823A, F826N and N827A mutants, all of which lost fusion activity and were detected by R1-5G2, we selected F826N and N827A mutants for subsequent studies.

### Generation and amplification of the scMP-12

Because development of scMP-12 is aimed at improving RVF vaccine safety, it is important to prevent the generation of infectious viruses in scMP-12-immunized hosts, as well as during scMP-12 preparation in cell culture. Hence, we tested several M RNA mutants, each encoding Gn/GcΔ5, with different combinations of fusion peptide mutations and chose an M RNA mutant encoding Gn/GcΔ5 with the F826N and N827A mutations (scMP-12 M RNA)(Fig. 1B) for scMP-12 preparation primarily due to its excellent genetic stability. BSR-T7/5 cells were co-transfected with three protein expression plasmids expressing the L, N, and Gn/Gc proteins, and three RNA expression plasmids encoding the L, scMP-12 M, and S-GFP RNAs. The GFP signal generated in scMP-12-infected cells facilitated the monitoring of scMP-12 replication. We also generated a VRP, an MP-12-based virus replicon particle (VRP) carrying only the L and S-GFP RNAs. Because other groups have reported the generation of a VRP (also called RVFV replicon particles) carrying the L and S-GFP RNAs derived from wt RVFV [44], [45], we refer to the wt virus-based VRP as VRPwt to distinguish between it and the MP-12-based VRP used in this study. MP-12 was rescued as previously described [31], and used as a positive control. Culture fluids from MP-12 samples were collected at 5 days post-transfection, while those from the scMP-12 and VRP samples were collected at 10 days post-transfection; these samples were defined as P0 samples.

**Figure 3:**
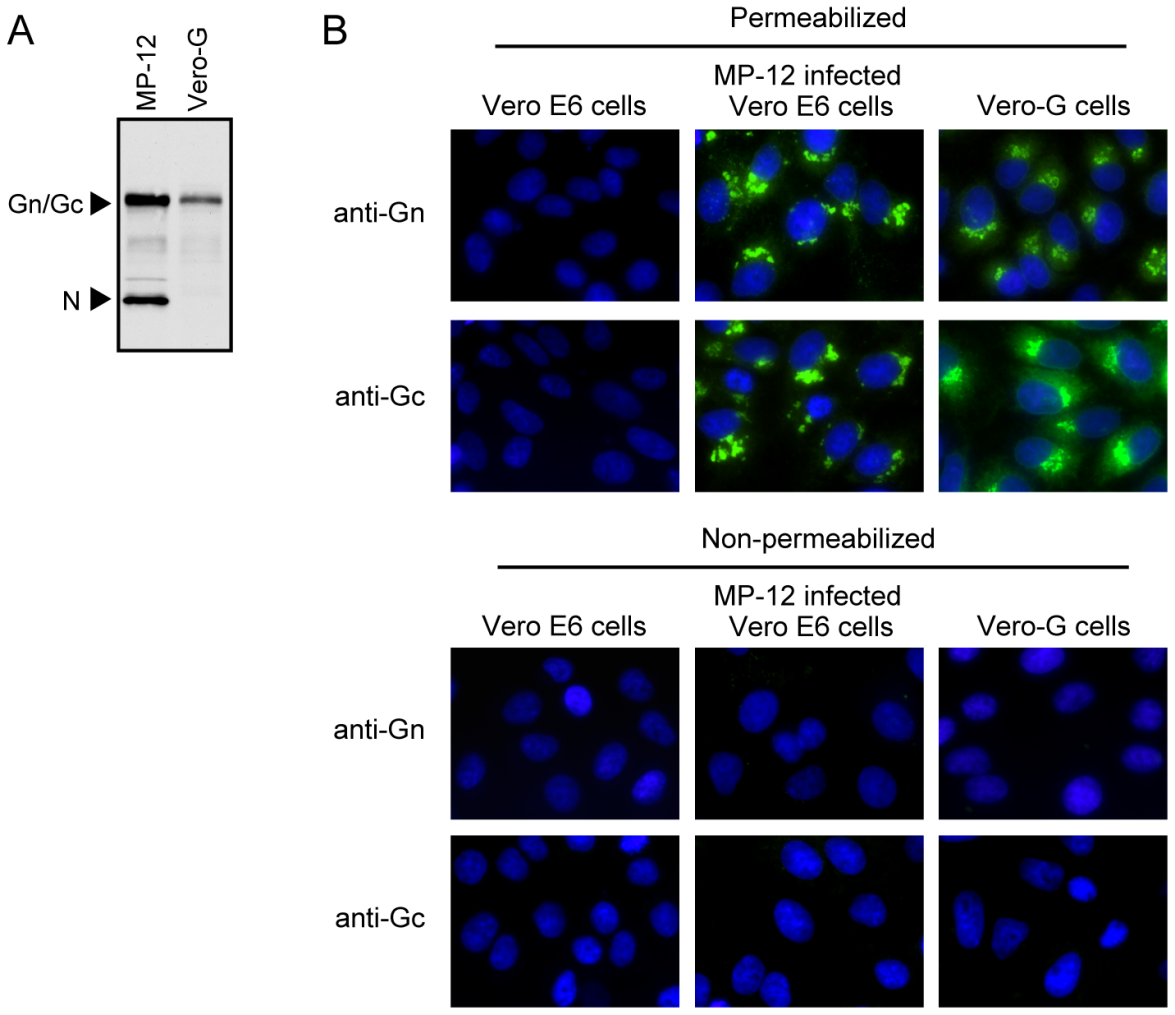
Expression of Gn/Gc in Vero-G cells. (A) Vero E6 cells were infected with MP-12 at an MOI of 1 and cell extracts were prepared at 12 h p.i. (MP-12). The same amounts of cell extracts were prepared from Vero-G cells (Vero-G). Both extracts were subjected to Western blot analysis by using anti-MP-12 antibody. (B) Subcellular localization of Gn/Gc in Vero-G cells. Mock-infected Vero E6 cells (Vero E6 cells), MP-12-infected Vero E6 cells and Vero-G cells were fixed, treated with Triton X-100 (permeabilized) or left without treatment (non-permeabilized) and stained with anti-Gn (Gn) or anti-Gc (Gc) monoclonal antibodies.

**Figure 4:**
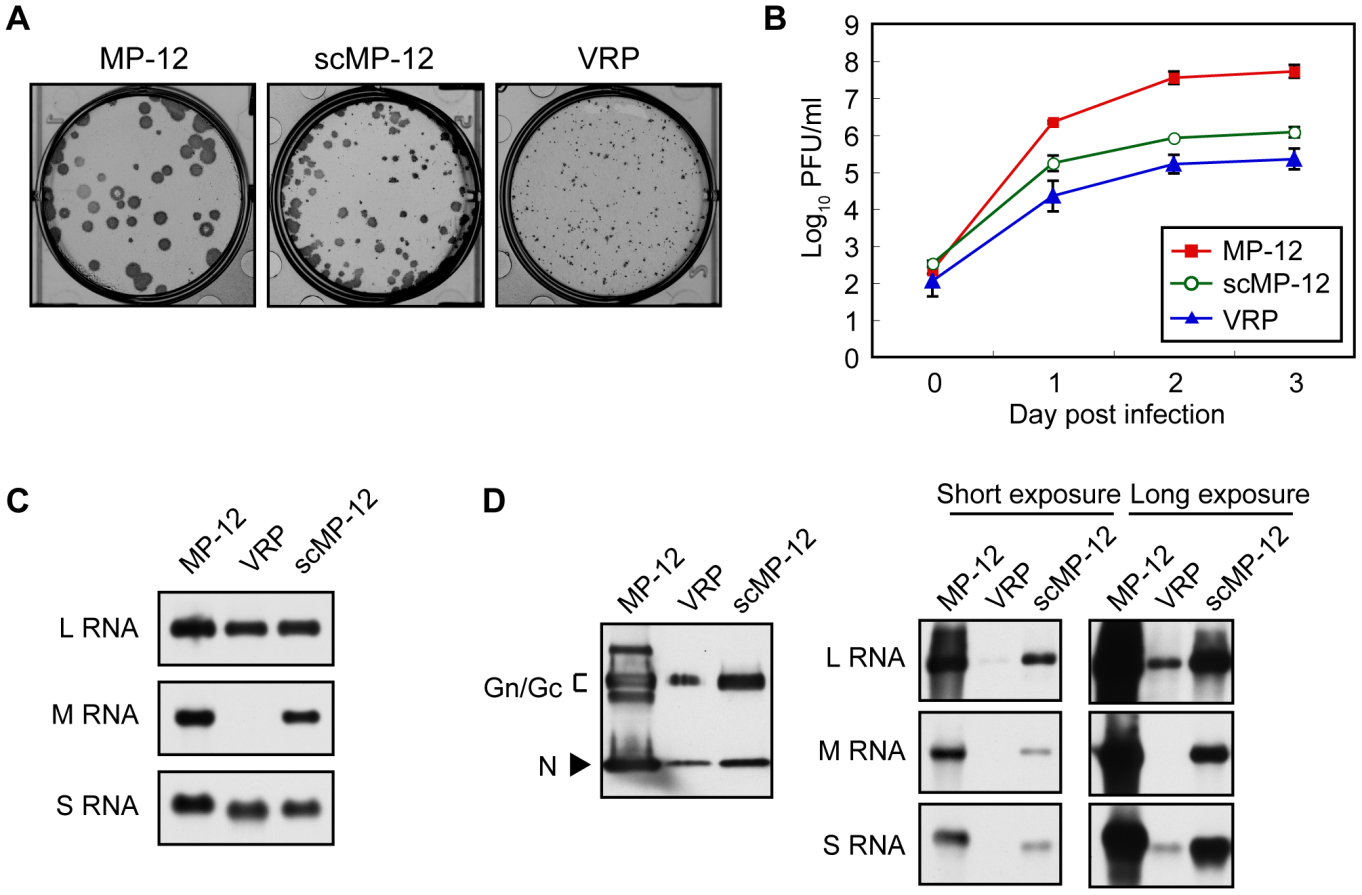
Replication of the scMP-12 in Vero-G cells. (A) Plaque formation of MP-12, scMP-12 and VRP in Vero-G cells. Vero-G cells were infected with MP-12, scMP-12, or VRP and overlaid with medium containing Tragacanth gum. After fixing the cells, plaques were stained with anti-N antibody and visualized using a Nova RED peroxidase substrate. (B) Growth kinetics of scMP-12, MP-12, and VRP in Vero-G cells. Vero-G cells were infected with scMP-12, MP-12, and VRP at an MOI of 0.05 and viral titers at 24, 48, 72, and 96 h p.i. were determined by plaque assay in Vero-G cells. The data are reported as mean titers with standard deviations of three independent experiments. (C) Vero-G cells were infected with MP-12, scMP-12, and VRP at an MOI of 0.05. Intracellular RNAs were harvested at 16 h p.i. and subjected to Northern blot analysis using RNA probes which hybridized with viral-sense L, M, or S RNA. (D) Vero-G cells were infected with MP-12, scMP-12, or VRP at an MOI of 0.02, and culture fluid was collected at 3 days p.i. The released particles were purified by sucrose gradient centrifugation and subjected to Western blot analysis using anti-MP-12 antibody (left panel). RNA samples corresponding to the samples in the left panel were subjected to Northern blot analysis using RNA probes that hybridized with viral-sense L, M, or S RNA (right panel).

To amplify and titrate the scMP-12 samples, we generated Vero-G cells stably expressing MP-12 Gn/Gc, and found the expression levels of Gn/Gc in Vero-G cells to be roughly one-fourth of the levels for MP-12-infected Vero cells at 12 h post-inoculation (p.i.) (Fig. 3A). Like MP-12-infected Vero E6 cells, Gn and Gc signals primarily accumulated in perinuclear regions of Vero-G cells (Fig. 3B). We independently inoculated the P0 samples of scMP-12 and VRP into Vero-G cells and obtained passage 1 (P1) samples after 10 days p.i. These P1 samples were predominantly used for subsequent studies. MP-12, scMP-12 and VRP formed large, medium and small plaques, respectively, in Vero-G cells, in which plaques were visualized by anti-N protein antibodies (Fig. 4A). Inoculation of MP-12, scMP-12 or VRP into Vero-G cells at a multiplicity of infection (MOI) of 0.05 showed efficient MP-12 replication with maximum titers ∼10^8^ PFU/ml at 3 days p.i. (Fig. 4B). scMP-12 replicated to ∼10^6^ PFU/ml at 2–3 days post-infection, whereas the titers of the VRP were roughly 5–10 times lower than those of scMP-12 (Fig. 4B). As expected, we observed efficient accumulation of the three viral RNA segments in Vero-G cells infected with MP-12 or scMP-12, and L and S-GFP RNAs in VRP-infected Vero-G cells (Fig. 4C). We purified the particles produced from Vero-G cells infected with scMP-12, MP-12 or VRP by sucrose gradient centrifugation. Western blot analysis of purified particles using anti-MP-12 antibody showed the presence of Gn/Gc and N proteins in all samples (Fig. 4D). The origin is unknown for two bands found, one that migrated more slowly and the other faster than the Gn/Gc of the MP-12 sample in the gel. Northern blot analysis of viral RNAs extracted from the purified particles showed packaging of three viral RNAs in MP-12 and scMP-12 samples and that of the L and S-GFP RNAs in the VRP sample (Fig. 4D); the abundance of each of the viral RNAs was roughly proportional to the titers of MP-12, scMP-12 and VRP at day 3 (Fig. 4B). These data show that the scMP-12 underwent efficient replication and amplification in Vero-G cells.

### Single-cycle replication property and genetic stability of the scMP-12

**Figure 5:**
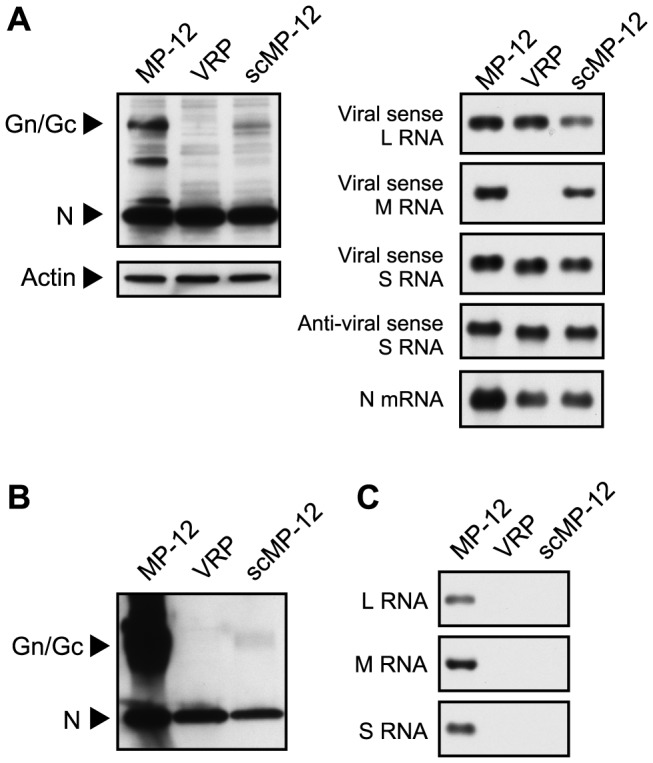
Viral gene expression and VLP production in scMP-12-infected naïve cells. (A) BHK cells were infected with MP-12, scMP-12, or VRP at an MOI of 0.05, and intracellular proteins and RNAs were harvested at 16 h p.i. Intracellular proteins were subjected to Western blot analysis using anti-MP-12 antibody or anti-actin antibody (left panel). RNA samples were subjected to Northern blot analysis by using RNA probes, each hybridizing with viral-sense L RNA, viral-sense M RNA, viral-sense S RNA, and both anti-viral sense S RNA and N mRNA (right panel). (B) BHK cells were infected with MP-12, scMP-12, or VRP at an MOI of 0.02, and culture fluids were collected at 3 days p.i. Released particles were purified by sucrose gradient centrifugation and subjected to Western blot analysis using anti-MP-12 antibody. (C) BHK cells were infected with MP-12, scMP-12 or VRP at an MOI of 0.05, and culture fluids were collected at 3 days p.i. The culture fluid was inoculated into fresh BHK cells, and intracellular RNAs were extracted at 12 h p.i. RNA samples were subjected to Northern blot analysis using RNA probes, which hybridized with viral-sense L, M, or S RNA.

To examine scMP-12 replication in naïve cells, we inoculated MP-12, scMP-12 or VRP into naïve BHK cells and examined the accumulation of viral proteins and RNAs (Fig. 5A). Efficient accumulation of the Gn/Gc and N proteins occurred in MP-12-infected cells. Accumulation of the N and Gn/Gc proteins also occurred in scMP-12-infected cells, with lower levels of Gn/Gc accumulation as compared to MP-12-infected cells. VRP-inoculated cells showed an accumulation of the N protein, but not the Gn/Gc protein. Northern blot analysis showed that the three viral RNAs replicated in MP-12-infected cells and in scMP-12-infected cells, and L and S-GFP RNAs replicated in VRP-infected cells. An RNA probe that specifically binds to anti-viral-sense S RNA clearly demonstrated N mRNA synthesis in these RNA samples (Fig. 5A, right panels). Thus, the scMP-12 underwent efficient viral RNA synthesis and viral protein accumulation in infected naïve cells.

We next purified the particles released from scMP-12-infected BHK cells by sucrose gradient centrifugation and detected viral proteins in the purified particles (Fig. 5B). The purified particles produced from MP-12-infected cells and VRP-infected cells served as a positive control and a negative control, respectively. Western blot analysis showed the production of MP-12 particles in the positive control by demonstrating the N and Gn/Gc proteins. No Gn/Gc signal was detected in the VRP sample, whereas the scMP-12 sample showed a low level of Gn/Gc signal. Both scMP-12 and VRP samples showed low levels of the N protein signal. Because synthesis of the Gn/Gc proteins did not occur in VRP-infected cells, it is highly unlikely that the N protein in the VRP sample represents released VRP. Continuous sucrose gradient centrifugation of culture fluid of MP-12-infected cells showed sedimentation of N protein with the purified virions as well as to lower sucrose density fractions [46], suggesting the release of N protein which is not associated with virus particles from infected cells. Furthermore, release of N protein not associated with viral envelope proteins was reported in studies of RVFV VRP [45] and Crimean-Congo hemorrhagic fever virus [47]. Hence, the N protein signal in the VRP sample most probably represents the N protein that was not associated with virus particles. Likewise, most of the N signal in the scMP-12 sample was probably derived from the non-VLP-associated N protein. Nonetheless, the Gn/Gc signal in the scMP-12 sample suggests the occurrence of low levels of VLP production from scMP-12-infected naïve cells. Inoculation of supernatant from MP-12-infected BHK cells, but not from scMP-12-infected BHK cells or VRP-infected BHK cells, into fresh BHK cells resulted in viral RNA synthesis (Fig. 5C), demonstrating that the VLP produced from scMP-12-infected naïve cells was not infectious.

To evaluate the genetic stability of scMP-12, we performed 10 serial passages of scMP-12 in Vero-G cells under three different conditions, as described in Materials and Methods, and tested the generation of infectious viruses that undergo multiple cycles of replication in naïve cells. Multiple cycles of the scMP-12 amplification in Vero-G cells resulted in an increase in the numbers of GFP-positive cells during incubation in each passage, whereas an increase in the numbers of GFP-positive cells did not occur after inoculation of any of the passage samples in Vero cells, suggesting the absence of infectious viruses in all of the passaged samples. Also plaque assays using Vero cells did not show the presence of infectious viruses in any of the samples. Sequence analysis of the PCR products of scMP-12 M RNA showed that scMP-12 retained the introduced mutations after 10 passages under the three different conditions. These results demonstrate that scMP-12 stably retained the introduced mutations.

### Testing the neurovirulence of the scMP-12

**Figure 6:**
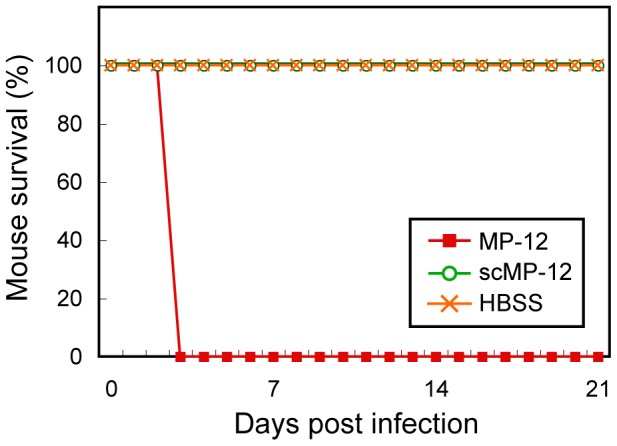
Neurovirulence of the scMP-12. Two-day-old CD-1 mice were intracranially inoculated with 10^4^ PFU of MP-12, scMP-12, or HBSS, and their survival was monitored for 21 days (MP-12, n = 10; HBSS, n = 10; scMP-12, n = 9).

We tested the neurovirulence of scMP-12 by intracranially inoculating 1.0×10^4^ PFU of scMP-12 into 2-day-old CD1 mice and monitoring for survival and clinical signs for 21 days p.i. As controls, HBSS and the same titer of MP-12 were inoculated. All MP-12 infected mice died by 3 days p.i., whereas all mice inoculated with scMP-12 or HBSS survived and did not show any clinical signs of disease (Fig. 6), demonstrating the absence of detectable levels of neurovirulence in scMP-12.

### Immunogenicity and protective efficacy of scMP-12

**Figure 7:**
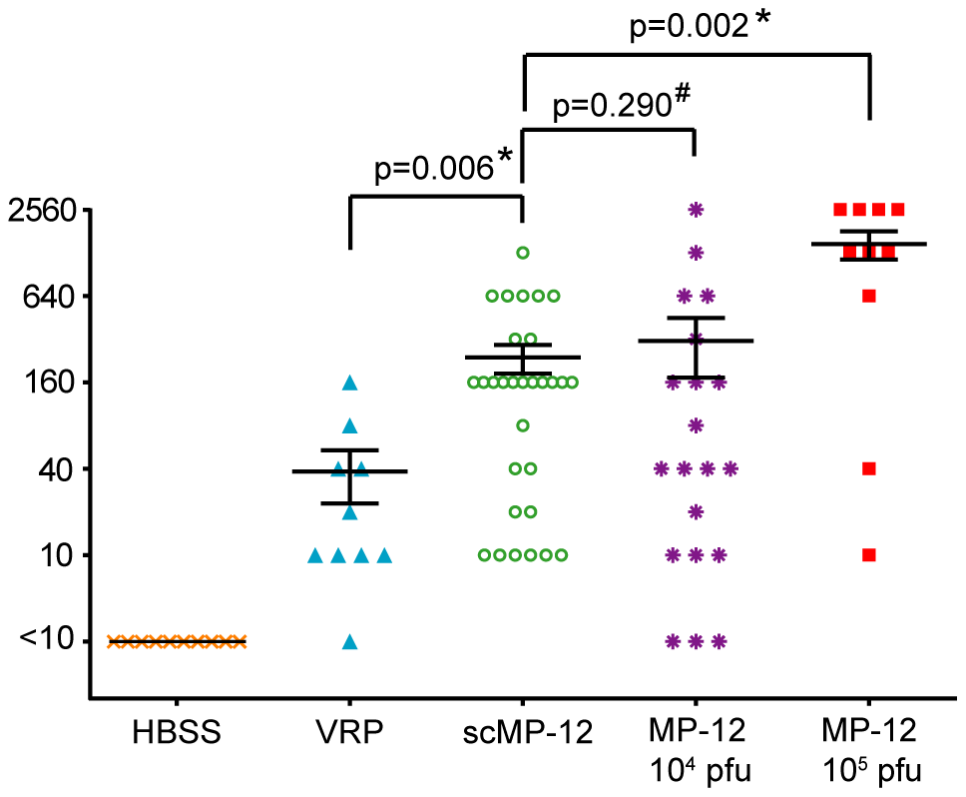
Immunogenicity of MP-12, scMP-12, and VRP. Five-week-old female CD1 mice were intramuscularly immunized with 10^4^ PFU of MP-12, 10^5^ PFU of MP-12, 10^5^ PFU of scMP-12, 10^5^ PFU of VRP or with HBSS. Thirty-six days later, blood was collected and subjected to PRNT80. The error bars represent standard deviations. A Mann-Whitney U test was performed for statistical analysis between PRNT80 titers (*, P<0.01; #, No significant difference). The error bars indicate the standard error of the mean. (10^4^ PFU of MP-12, n = 20; 10^5^ PFU of MP-12, n = 10; 10^5^ PFU of scMP-12, n = 30; 10^5^ PFU of VRP, n = 10; and HBSS, N = 10).

We intramuscularly inoculated 5-week-old female CD1 mice once with 10^5^ PFU of scMP-12 and determined the PRNT80 titers at 36 days p.i. As controls, mice were inoculated with 10^5^ PFU of VRP, 10^5^ PFU of MP-12, 10^4^ PFU of MP-12, or HBSS (Fig. 7). HBSS-inoculated mice had no detectable neutralizing antibody titers, while mice inoculated with 10^5^ PFU of MP-12 and 10^4^ PFU of MP-12 had a mean PRNT80 titer of 1∶1,477 and 1∶310, respectively. The mean PRNT80 titers of the mice immunized with 10^5^ PFU of scMP-12 and 10^5^ PFU of VRP were 1∶238 and 1∶38, respectively; the difference in the PRNT80 titers was statistically significant. Thus, the mice immunized with 10^5^ PFU of scMP-12 elicited neutralizing antibody titers that were statistically higher than those immunized with 10^5^ PFU of VRP and were comparable to those immunized with 10^4^ PFU of MP-12.

**Figure 8:**
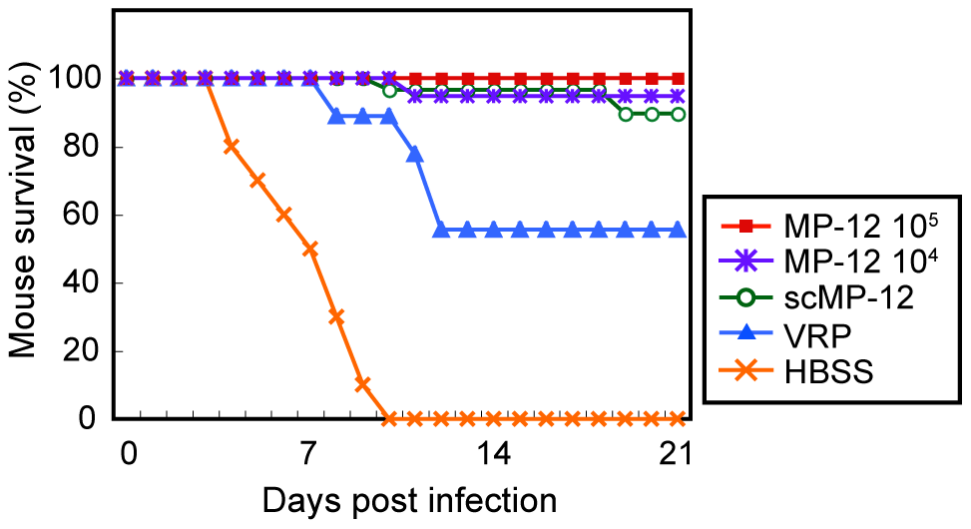
Protection efficacy of scMP-12. Five-week-old CD1 mice were intramuscularly immunized with 10^4^ PFU of MP-12, 10^5^ PFU of MP-12, 10^5^ PFU of scMP-12, and 10^5^ PFU of VRP or with HBSS. Forty days after vaccination, immunized mice were challenged with 10^3^ PFU of the wt ZH501 strain of RVFV (10^4^ PFU of MP-12, n = 19; 10^5^ PFU of MP-12, n = 10; 10^5^ PFU of scMP-12, n = 29; 10^5^ PFU of VRP, n = 9; and HBSS, N = 10). The mice were observed and survival recorded for 21 days after challenge.

We next tested whether scMP-12 immunization protects mice from wild-type RVFV challenge. Five-week-old female CD1 mice were intramuscularly inoculated once with 10^5^ PFU of scMP-12, 10^5^ PFU of VRP, 10^5^ PFU of MP-12, 10^4^ PFU of MP-12, or HBSS. At 40 days post-immunization, the mice were challenged subcutaneously with 1.0×10^3^ PFU of the ZH501 strain of RVFV and their survival was monitored for 21 days p.i. (Fig. 8). All HBSS-inoculated mice died by 10 days p.i., whereas all mice immunized with 10^5^ PFU of MP-12 survived. Most of the mice immunized with 10^4^ PFU of MP-12 or 10^5^ PFU of scMP-12 survived, yet 1 of the 19 MP-12-immunized mice died at day 11, and 3 of the 29 scMP-12-immunized mice died, one at day 10 and two at day 19, respectively. In contrast, 45% of the VRP-immunized mice died by day 12 p.i., demonstrating that scMP-12 immunization protected most of the mice from wt RVFV challenge, and scMP-12-induced protection was better than the VRP-induced protection.

**Figure 9:**
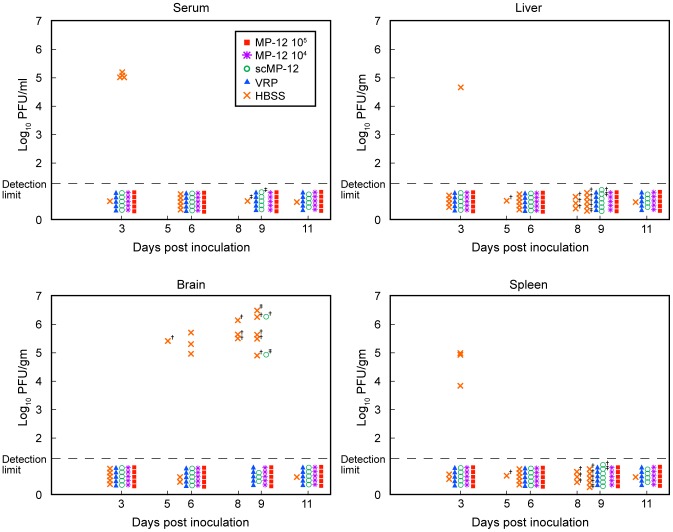
Titers of wt ZH501 in the serum, liver, spleen and brain of immunized mice. Five-week-old CD1 mice were intramuscularly immunized with 10^4^ PFU of MP-12, 10^5^ PFU of MP-12, 10^5^ PFU of scMP-12, 10^5^ PFU of VRP or with HBSS. Forty days after vaccination, immunized mice were challenged with 10^3^ PFU of the wt ZH501 strain and virus titers in serum, liver, spleen and brain were determined at 3, 6, 9 and 11 days post-challenge. †, samples were obtained from dead animals; ‡, samples were obtained from moribund animals. Each icon represents a single animal.

To study the extent to which scMP-12-induced immune responses suppressed wt virus replication upon challenge, HBSS-inoculated mice and mice immunized once with 10^5^ PFU scMP-12, 10^5^ PFU VRP, 10^5^ PFU MP-12, or 10^4^ PFU MP-12 were challenged with the ZH501 strain of RVFV, as described above, and the virus titers in serum, liver, spleen and brain were determined at days 3, 6, 9 and 11 post-challenge (Fig. 9). At day 3 p.i., 4 out 5 HBSS-inoculated mice had >10^5^ PFU/ml of viremia, and one and three mice showed virus replication in the liver and the spleen, respectively. Efficient virus replication in the brain also occurred in HBSS-inoculated mice from days 5 to 9 p.i. In contrast, mice immunized with VRP or MP-12 showed neither viremia nor virus replication in the liver, spleen or brain. scMP-12 immunization also prevented viremia and virus replication in the liver and spleen, while two mice, one having no detectable PRNT80 titer and the other having a PRNT80 titer of 1∶20, showed virus replication in the brain at day 9 p.i.

## Discussion

By using a membrane fusion assay and newly established Vero-G cells, we generated scMP-12 and tested its potential as a safe and immunogenic RVFV vaccine. scMP-12 amplified efficiently in Vero-G cells and stably retained the introduced mutations in ten serial passages in this cell line under three different experimental conditions. In infected naïve cells, scMP-12 underwent efficient viral RNA synthesis and accumulated viral proteins, including Gn/Gc, and produced low levels of non-infectious VLPs. The scMP-12 did not show any sign of neurovirulence after intracranial inoculation into 2-day-old mice, demonstrating excellent safety. scMP-12 immunization in mice induced neutralizing antibodies, whose titers were higher than those in VRP-immunized mice, and protected most of them from wt RVFV challenge by suppressing viremia and wt RVFV replication in the liver and the spleen. Taken together, we consider that scMP-12 has an excellent potential to be developed as a novel safe RVFV vaccine.

We examined effects of mutations within the putative fusion peptide for membrane fusion (Fig. 2). The crystal structure of RVFV Gc suggested that V828 and hydrophobic residues W821 and F826 within the putative fusion peptide serve as a membrane anchor during the pre-fusion step [41]. By substituting the hydrophobic residues for hydrophilic residues in the putative fusion peptide, we experimentally demonstrated that an F826N mutation, but not V828N mutation, abolished membrane fusion. Anti-Gc monoclonal antibody did not recognize Gc carrying W821N, and possibly a van der Waals interaction between W821 and F826 was disrupted by this mutation, leading to Gc structural alteration [41]. C825 is highly conserved among Phleboviruses (Fig. 2C), and the C825A mutant was defective for the fusion function. Because C825 is involved in a disulfide bond in Gc [41], and the anti-Gc monoclonal antibody did not recognize the C825A mutant, a lack of fusion function in this mutant was probably due to the structural alteration of Gc. Because other Phleboviruses also encode the ER retrieval signal in the Gc cytoplasmic tail, development of similar membrane fusion assays for other Phleboviruses would be possible. Recently, others also reported the utility of the RVFV membrane fusion assay that uses a plasmid transfection method [48]. Experiments using such fusion assays, which employ a conventional plasmid transfection, will be valuable for further understanding of the membrane fusion mechanism in Phleboviruses, and identification and evaluation of antivirals that suppress viral membrane fusion activity [48].

The data that RVFV spread can be prevented by effective vaccination of animals and humans [1] and that neutralizing antibodies, the majority of which recognize Gn/Gc protein, play a critical role in protection [12]–[20] led to development of several different types of RVFV vaccine candidates that primarily aim to elicit high titers of neutralizing antibodies. Formalin-inactivated RVFV vaccine requires several immunizations to induce and maintain protective immunity [49], [50]. In contrast, several attenuated RVFV mutants, including MP-12, MP-12-derived mutants carrying a modified cellular gene in place of the NSs gene [51], and a wt RVFV-derived avirulent mutant lacking NSs and NSm genes, both of which are viral virulence factors [52]–[55], demonstrated excellent protective immunogenicity against wt RVFV after a single immunization of animals [56]. Examples of other vaccine candidates are VLPs [57]–[59], recombinant vaccinia viruses encoding Gn and Gc proteins [29], alphavirus encoding the Gn protein [60], alphavirus replicon encoding the Gn protein [61], and a soluble ectodomain of the Gn protein [62]. Most of these vaccine candidates have used multiple dose immunization protocols to confer complete protection to immunized rodents against wt RVFV challenge. Single immunization of mice with scMP-12 (Fig. 8), VLP expressing low levels of viral N protein in infected cells [59] or VRPwt expressing both L and N proteins in inoculated cells [44], [63] showed good protection of the immunized mice from lethal wt RVFV challenge. This finding may imply that the expression of the N protein and probably also the L protein in immunized animals facilitated development of strong protective immune responses. In addition, viral-replicating, single-stranded RNA and the incoming RNA virus nucleocapsids activate the innate immune system through interaction with the host pattern recognition receptor, e.g. RIG-I [64]–[69], and potentiates the adaptive immune responses [70]. Moreover, viral RNAs in virus particles have an adjuvant effect for augmenting host-adaptive immune responses through a Toll-like receptor 7 signaling pathway in dendritic cells [71], [72]. Therefore, it is likely that incoming nucleocapsids of scMP-12, intracellular viral RNAs accumulated in scMP-12-infected naïve cells, and viral RNAs in the released VLPs all contributed to enhancement of the host immune response, making scMP-12 highly immunogenic. Importantly, scMP-12 was more immunogenic than VRP (Fig. 7), and the scMP-12-immunized mice were protected from wt RVFV challenge more efficiently than the VRP-immunized mice (Fig. 8); hence, the expression of Gn/Gc in cells supporting scMP-12 replication and viral RNA containing VLPs produced by cells in which scMP-12 replicated augmented the protective immune response.

A lack of neurovirulence and the characteristic single-cycle replication property of scMP-12 demonstrate that scMP-12 is superior to MP-12 in safety, as MP-12 killed all of the 2-day-old mice following intracranial inoculation, whereas scMP-12 was less immunogenic than MP-12; neutralizing antibody titers in mice immunized with 10^5^ PFU of scMP-12 were comparable to those immunized with 10^4^ PFU of MP-12 and lower than those immunized with 10^5^ PFU of MP-12. Improvement of scMP-12 immunogenicity may be possible by generating a scMP-12 variant that produces a high abundance of VLPs following scMP-12 replication. Because substitution of several histidines in RVFV Gc with alanine inhibits membrane fusion activity but does not interfere with virion assembly [73], the efficient production of noninfectious VLPs may occur in cells supporting replication of scMP-12 variants carrying some of these mutations.

The finding of efficient scMP-12 amplification in Vero-G cells suggests that a scMP-12-based vaccine stock can be prepared in Vero-G cells or their equivalent without plasmid transfection, thereby allowing the production costs of the scMP-12-based vaccine to be comparable to those for MP-12. scMP-12 and VRP produced plaques in Vero-G cells, showing the utility of Vero-G cells for easy titration and characterization of RVFV mutants lacking functional Gn/Gc proteins. We noted that MP-12 replicated roughly 10 times better in Vero-G cells than in Vero E6 cells (Fig. 4B and [31]), which led us to suggest that higher levels of intracellular Gn/Gc accumulation augments MP-12 production. Likewise, an increase in the abundance of intracellular Gn/Gc in scMP-12-replicating cells may also enhance scMP-12 titers. Hence, the development of another Vero cell clone, in which expression levels of Gn/Gc are comparable to those in MP-12-infected Vero cells, would contribute to mass immunization programs using an scMP-12-based vaccine. The absence of infectious virus after 10 serial passages of scMP-12 in Vero-G cells under three different conditions demonstrated that homologous RNA recombination that can eliminate the mutations in scMP-12 M RNA did not occur between replicating scMP-12 M RNA and expressed mRNA encoding Gn/Gc in Vero-G cells, further indicating the utility and safety of Vero-G cells for preparation of the scMP-12-based vaccine. Lastly, we found that scMP-12 replicated ∼10 times better than did the VRP in Vero-G cells (Fig. 4). These data were consistent with the notion that M RNA serves important roles in viral RNA co-packaging [32].

Expression of GFP from the S-GFP RNA of scMP-12 facilitated easy monitoring of scMP-12 replication and generation of infectious viruses in scMP-12 preparations. VRPwt also used S-GFP-type RNA for easy monitoring of VRPwt replication [44], [45]. However, vaccines encoding a foreign reporter gene, such as GFP, may not be appropriate for human use. Therefore, before we can develop a scMP-12-based human vaccine, it is necessary to test the replication competence, safety, and immunogenicity of scMP-12-based vaccine candidates lacking the NSs gene or of those carrying RVFV Clone 13-type S RNA lacking ∼70% of the NSs gene [74].

Our study was primarily aimed at the development of a safe and immunogenic human RVF vaccine, yet scMP-12 may be further developed as a veterinary vaccine. Others have reported that MP-12 is teratogenic in some cases [75]. Considering that scMP-12 only undergoes a single cycle of replication, it is unlikely cause disease in immunized animals. Vaccines that are compatible with a differentiation of infected and vaccinated animals (DIVA) are suitable for use as animal vaccines. Examples of replication-competent RVF DIVA vaccine candidates are RVFV Clone 13 lacking ∼70% of the NSs gene [74], MP-12 lacking NSm, which elicited high titers of neutralizing antibodies in sheep and calves [76], and wt RVFV-derived mutant virus lacking NSm and NSs, which induced protective immunity in immunized sheep [56]. The data that scMP-12, which lacks an NSs gene, protected immunized mice from wt RVFV challenge (Fig. 8) and that VRPwt, which also lacks an NSs gene, can induce protective immunity in sheep [63] indicate a potential for a scMP-12-based DIVA vaccine to reduce the incidence of RVF among humans and animals and to control this important pathogen [8].
